# Long noncoding RNAs (lncRNAs) and the molecular hallmarks of aging

**DOI:** 10.18632/aging.100710

**Published:** 2014-12-22

**Authors:** Ioannis Grammatikakis, Amaresh C. Panda, Kotb Abdelmohsen, Myriam Gorospe

**Affiliations:** Laboratory of Genetics, National Institute on Aging-Intramural Research Program, NIH, Baltimore, MD 21224, USA

**Keywords:** long noncoding RNAs, senescence-associated lncRNAs, epigenetic control by lncRNAs, lncRNA regulation of post-transcriptional gene expression, lncRNA influencing aging phenotypes

## Abstract

During aging, progressive deleterious changes increase the risk of disease and death. Prominent molecular hallmarks of aging are genomic instability, telomere attrition, epigenetic alterations, loss of proteostasis, cellular senescence, stem cell exhaustion, and altered intercellular communication. Long noncoding RNAs (lncRNAs) play important roles in a wide range of biological processes, including age-related diseases like cancer, cardiovascular pathologies, and neurodegenerative disorders. Evidence is emerging that lncRNAs influence the molecular processes that underlie age-associated phenotypes. Here, we review our current understanding of lncRNAs that control the development of aging traits.

## INTRODUCTION

Aging is associated with a progressive deterioration in the function of cells, tissues, and organs. This decline in function eventually leads to age-related declines in physiologic function, such as loss of muscle mass, declining blood flow, impaired immune recognition, decreased ability to utilize energy, diminished cognitive function, and reduced ability to respond to stress stimuli. These age-related declines culminate in the onset of age-associated diseases like sarcopenia, cardiovascular disease, cancer, obesity, diabetes, neurodegenerative diseases, and many other pathologies. Recently, Lopez-Otín et al [1] cataloged the molecular processes that decline with advancing age and underlie the phenotypes of aging. They include epigenetic changes, loss of telomere function, declining protein homeostasis, increased cellular senescence, depletion of the stem cell pool, and altered intercellular communication.

The phenotypic changes that characterize the aging process are governed by specific alterations in the pools of expressed proteins. Therefore, there is heightened interest in understanding the mechanisms that drive age-associated gene expression programs. These processes are mainly directed by proteins that bind DNA and RNA, as well as by a variety of noncoding (nc) RNA, both short ncRNAs (mainly microRNAs) and long (lncRNAs). Together, this vast and heterogeneous group of factors affects aging by controlling gene expression transcriptionally and post-transcriptionally in myriad different ways. The influence of transcription factors, RNA-binding proteins, and microRNAs in age-associated processes has been reviewed elsewhere [2-4]. Some lncRNAs have been studied for decades, but the broad range of expressed lncRNAs and their impact on protein expression programs has only come into view in recent years. LncRNAs modulate gene expression patterns at all levels: transcriptional, post-transcriptional, and post-translational [5-7]. Through their impact on the type and abundance of proteins, lncRNAs affect key cellular processes such as proliferation, differentiation, quiescence, senescence, the cellular response to stress and immune agents, and many others cellular functions relevant to the biology of aging. In this review, we describe and discuss the emerging impact of lncRNAs on these processes and their possible implications in senescence, aging and age-related pathologies.

**LncRNAs.** LncRNAs vary widely in size, genomic localization, and biogenesis. They can be expressed from intergenic regions (lincRNAs), from the opposite strand of mRNAs (antisense lncRNAs), from vestigial genes that lost their coding potential (pseudogene-encoded lncRNAs), from introns of annotated genes (long intronic ncRNAs), or from the promoter regions of coding mRNAs (promoter-associated lncRNAs); they can also be generated by the splicing machinery (circular RNAs) [8].

**Figure 1:**
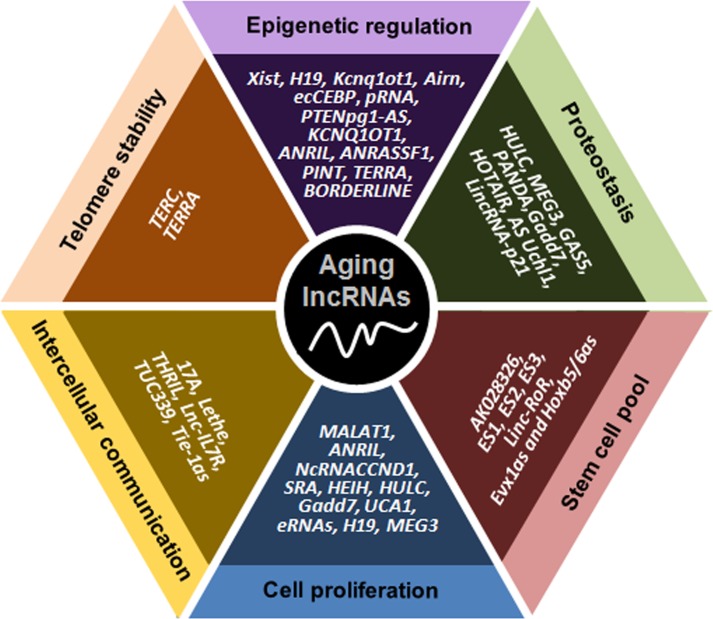
Schematic representation of lncRNAs affecting six major molecular traits of aging The lncRNAs indicated have been implicated in controlling telomere length, epigenetic gene expression, proteostasis, the pool of stem cells, cell proliferation and senescence, and communication among cells.

LncRNAs can be classified according to their molecular mechanism of action. Some nuclear lncRNAs can regulate gene expression epigenetically by recruiting chromatin-modification factors to activate or inactivate different loci [9, 10]. LncRNAs can also serve as transcriptional regulators by assembling transcriptional activators and repressors to modulate transcription initiation by RNA polymerase II [10, 11]. Other lncRNAs can function in nuclear compartmentalization and help maintain nuclear structures such as nuclear speckles, paraspeckles, and interchromatin granules [11]. In addition, lncRNAs can regulate gene expression post-transcriptionally by base-pairing with mRNAs to modulate their translation and/or stability [12-14] and by interfering with RNA-binding proteins to influence splicing and translation [14-16]. Competing endogenous RNAs (ceRNAs) and circular RNAs are stable lncRNAs that accumulate in large numbers and modulate gene expression in different ways, including as decoys or sponges for microRNAs [17, 18]. Finally, some lncRNAs have been shown to control protein turnover by facilitating ubiquitination [7] (Figure 1, Table 1).

**Table 1:** LncRNAs affecting molecular traits of aging

LncRNAs	Function in Aging Traits	Comments	Refs.
**LncRNAs controlling telomere function**			
***TERC***	Promotion of telomere extension	Terc KO mice age prematurely	[20]
***TERRA***	Suppression of telomere extensionHeterochromatin	High TERRA triggers senescenceModulates telomeric structure	[23, 24][89]
**LncRNAs controlling epigenetic changes**			
***Xist***	DNA methylation	Declines with senescence	[34]
***H19***	DNA methylation Cell division cycle	Increases in some cancers Enhances proliferation	[39] [37-40]
***Kcnq1ot1***	DNA methylation, cell division	Implicated in cancer and diabetes	[43]
***Airn***	DNA methylation	Controls IGF signaling pathway	[45]
***ecCEBP***	DNA methylation	Affects senescence TFs C/EBP	[49,50]
***pRNA***	DNA methylation	Linked to reduced rRNA in AD	[55,56]
***PAPAS***	Histone methylation	Chromatin compaction at rDNA loci	[58]
***PTENpg1-AS***	DNA methylation	Modulates PTEN expression	[59]
***TARID***	DNA methylation	Triggers expression of TCF21	[65]
***KCNQ1OT1***	Histone modifications	Blocks Kcnq1 (linked to age, CVD)	[73,74]
***NeST***	DNA methylation	Increases IFN-γ upon infection	[76]
***ANRIL***	Histone modifications	Controls p16 levels (senescence, aging)	[77]
***ANRASSF1***	Histone modifications	Recruits PRC2 to RASSF1A promoter	[79]
***PINT***	Histone modifications	Recruits PRC2, elicits p53 actions	[80-82]
***BORDERLINE***	Heterochromatin	Processed into brdrRNAs by Dicer	[90]
**LncRNAs affecting proteostasis**			
***HULC***	Autophagy	Inhibits apoptosis, promotes autophagy	[97]
***MEG3***	Autophagy Growth arrest	Suppresses MDM2, upregulates p53 Blocks apoptosis	[99] [100]
***7SL***	Autophagy Protein trafficking	Suppresses p53 Competes with HuR, linked to senescence	[14] [14]
***GAS5***	Protein trafficking	Binds GR, linked to cognitive decline	[102]
***PANDA***	Protein trafficking	Inducible by p53 upon DNA damage	[104,105]
***Gadd7***	Protein trafficking	Binds TDP-43, modulates Cdk6 levels	[107]
***HOTAIR***	Protein abundance	Up in senescence, ubiquitination	[7]
***AS Uchl1***	Protein abundance	Induces senescence, upregulated in PD	[110,111]
***LincRNA-p21***	Protein abundance	Represses translation of cancer proteins	[13]
**LncRNAs modulating stem cell function**			
***AK028326, AK141205***	Regulation of stem cell TFs	Control function of mouse ESC TF	[123]
***ES1, ES2, ES3***	Regulation of stem cell TFs	Regulated by human ESC, iPSC factors	[129]
***linc-RoR***	Regulation of stem cell TFs	Regulates hESCs reprograming	[130]
***Evx1as, Hoxb5/6as***	Histone methylation in stem cells	Associated with ES cell pluripotency	[133]
**LncRNAs controlling proliferation and senescence**			
***MALAT1***	Cell division cycle	Inhibits senescence, promotes division	[141]
***ANRIL***	Cell division cycle	Prevents expression of p15 and p16	[144]
***NcRNA_CCND1_***	Cell division cycle	Inhibits transcription of CCND1 gene	[119]
***SRA***	Cell division cycle	Inhibits senescence cdki p21, p27	[146,147]
***HEIH***	Cell division cycle	Inhibits senescence cdki p21, p16, p27	[149]
***HULC***	Cell division cycle	Inhibits senescence cdki p18	[152]
***Gadd7***	Cell division cycle	Binds TDP-43, lowers Cdk6 mRNA	[107]
***UCA1***	Cell division cycle	Inhibits senescence cdki p27	[156]
***eRNAs***	Cell division cycle	p53-regulated, affect senescence, aging	[161]
***7SL***	Cell division cycle	Lowers p53 levels, inhibits senescence	[14]
**LncRNAs governing intercellular communication**			
***17A***	Modulates inflammation	Upregulated in AD, linked to GPRs	[176]
***Lethe***	Modulates inflammation	Induced by TNFα, inhibits NF-κB	[178]
***THRIL***	Modulates inflammation	Induced by TNFα, interacts with hnRNPL	[120]
***Lnc-IL7R***	Modulates inflammation	Regulates LPS-mediated inflammation	[180]
***TUC339***	Transported in exosomes	Controls cell proliferation, tumor growth	[192]
***Tie-1as***	Transported in exosomes	Targets tie-1, affects endothelial junctions	[194]
***Linc-RoR***	Transported in exosomes	Contributes to chemoresistence in hepatocellular carcinoma cells	[195]

The table lists potential age-associated lncRNAs (column 1), the cellular and molecular processes they regulate (column 2), and specifics of their expression and/or function (column 3). AD, Alzheimer's disease; cdki, cyclin-dependent kinase inhibitor; CVD, cardiovascular disease; GR, glucocorticoid receptor; PD, Parkinson's disease; TF, transcription factor.

### LncRNAs that modulate telomere length

Telomeres are structures that protect the ends of chromosomes against damage. Cellular senescence is generally associated with a gradual shortening of telomere length [19]. In order for DNA replication to take place on the telomeres, a specific polymerase, telomerase reverse transcriptase (TERT), is required to extend telomeres to preserve their lengths [1]. The length of telomeres is regulated by the telomerase ribonucleoprotein complex that contains the protein TERT and the lncRNA *TERC* (telomerase RNA component), as well as by the telomeric repeat-containing RNA lncRNA *TERRA*.

***TERC.*** The essential RNA component of the telomerase enzyme complex, lncRNA TERC, has been directly implicated in the maintenance of telomere length and thus the prevention of premature senescence and aging. In support of this function, TERC-deficient mice displayed short telomeres, chromosomal instability, and premature aging [20]. TERC serves as a template for the synthesis of telomeric repeats and acts as a scaffold that brings the protein subunits of telomerase together with other accessory proteins associated with the complex. Additionally, TERC was shown to have a catalytic function in the process of adding telomere repeats [21].

***TERRA.*** While TERC promotes and maintains telomere length, the lncRNA TERRA suppresses telomere elongation. TERRA transcripts are transcribed from telomeres by RNA polymerase II, an unexpected discovery, since telomeres were long believed to be transcriptionally silent, and have variable lengths ranging between 100 and >9000 nt in mammals [22]. The suppression of telomeric RNA elongation is linked to the presence of numerous copies of the telomere UUAGGG repeat in the TERRA transcript, spanning ~200 nt [23], which renders TERRA a high-affinity ligand (and hence a competitive inhibitor) for TERT [24].

Abnormal expression of TERRA may contribute to premature senescence and aging. For instance, mutation in the gene DNA methyltransferase 3B (*DNMT3B*) leads to hypomethylation of the telomeric region and elevated levels of TERRA. These changes result in the syndrome ICF (immunodeficiency, centromeric instability, and facial dysmorphism), in which fibroblasts exhibit premature senescence linked to the suppression of telomere elongation [25]. TERRA is also involved in the removal of 3′G overhangs of uncapped telomeres during DNA damage-induced senescence [23]. Although the loss of telomeres is believed to accelerate senescence, telomerase-deficient cells can avoid senescence by a unique mechanism that requires TERRA during telomere recombination [26]. Early in S phase, the levels of TERRA decline, rising again at the end of S phase and thereby displacing hnRNPA1 from the telomeres and recruiting the single-stranded DNA-binding protein POT1 (protection of telomeres 1). Thus, while TERRA suppresses telomere elongation, it can also protect telomere ends [27].

### LncRNAs associated with epigenetic alterations in aging and senescence

Epigenetic changes modulate gene expression in a variety of processes that characterize age-associated pathology and physiology [28, 29]. In this section, we review the involvement of lncRNAs in epigenetic changes including DNA methylation, histone modification, and heterochromatin formation.

#### DNA methylation

A global decline in DNA methylation during aging and cellular senescence is well documented [30]. However, advancing age also leads to hypermethylation of several genes such as tumor suppressors and genes targeted by Polycomb group proteins in embryonic stem cells [31]. Several lncRNAs that contribute to the regulation of DNA methylation in the context of senescence and aging have been identified.

***Xist***. Responsible for imprinting and hence silencing of the X chromosome in females (to compensate for the dosage effect in males), *Xist* levels decline in senescent cells [32, 33, 34]. However, its specific function in senescence has not been described yet.

***H19***. The lncRNA H19 has been shown to play a role in embryonic development and growth [35]. It controls imprinting of a conserved cluster of genes that contains H19 itself and the insulin-like growth factor 2 (IGF2). This regulatory effect is mediated by the interaction of H19 with the methyl-CpG–binding domain protein 1 (MBD1) to form a ribonucleoprotein complex (H19-MBD1) that interacts with and recruits histone lysine methyltransferases and represses gene expression [36]. Both IGF2 and H19 are implicated in growth, proliferation, cell cycle, apoptosis, and aging [37-40]. Loss of imprinting of the IGF2-H19 locus during aging was observed in normal human prostate tissues leading to enhanced expression of IGF2, H19, and other genes in this locus. H19 levels were further elevated in old prostate tissues [41]. Loss of imprinting increases the levels of IGF2, which is associated with age-related diseases including cancer [42].

***Kcnq1ot1***. During mouse embryonic development, expression of the lncRNA *Kcnq1ot1* leads to transcriptional gene silencing by the recruitment of DNA methyltransferases (DNMTs) to the paternal allele of the *Kcnq* locus. The promoter of *Kcnq1ot1* is methylated at the maternal allele; the ensuing suppression of *Kcnq1ot1* expression allows gene expression of genes on the *Kcnq* locus relevant to age-associated diseases like type 2 diabetes and cancer [43]. One of the genes within this locus is the cyclin-dependent kinase (cdk) inhibitor and tumor suppressor *CDKN1C*, encoding P57^KIP2^ [44]. Thus, *Kcnq1ot1* can affect aging and senescence through its impact upon the cell cycle.

***Airn***. The lncRNA *Airn* is transcribed in antisense orientation to the maternally expressed *Igf2r* gene and controls *Igf2r* mRNA expression by silencing its transcription in *cis*. Interestingly, it is not the *Airn* lncRNA *per se*, but rather *Airn* transcription what drives methylation changes and *Igf2r* silencing [45]. *Airn* is likely involved in aging and senescence through its effect on IGF2R, since senescent cells show enhanced IGF2R expression compared to proliferating cells and IGF2R is implicated in longevity [46, 47]; however, a role for *Airn* in these age-related processes has not been studied directly.

***ecCEBP***. The lncRNA *ecCEBP* was also found to recruit DNMT1 in order to regulate local DNA methylation and silence the *CEBP* gene [48]. The encoded protein, CAAT enhancer-binding protein (C/EBP), influences cell cycle progression by interacting with and inhibiting kinases cdk2 and cdk4, triggering growth arrest [49]. The levels of several C/EBP family members, which are key regulators of adipogenesis, decline with age [50]. Recently, age-associated changes in C/EBP proteins were shown to cause severe liver injury and accelerated liver proliferation after treatment with CCl_4_ (carbon tetrachloride) [51], while C/EBPγ suppressed senescence and inflammatory gene expression by heterodimerizing with C/EBPβ [52]. Together, these findings indicate that the lncRNA *ecCEBP* regulates C/EBP expression and highlight its putative role in the age- and senescence-dependent changes in C/EBP abundance.

***pRNA***. The lncRNA *pRNA* regulates transcription of ribosomal RNA (rRNA) by interacting with DNA at the target site of the transcription factor TTF1; the resulting DNA-RNA triplex is specifically recognized by the DNA methyltransferase DNMT3b [53]. Accelerated rDNA methylation was observed in senescent Werner syndrome fibroblasts [54], although the possible involvement of *pRNA* in this process has not been examined. rRNA levels are selectively reduced with brain aging and this reduced ribosomal activity may contribute to Alzheimer disease (AD) [55, 56], although *pRNA* has not yet been implicated in the age-related changes in rRNA.

***PAPAS***. The heterogeneous population of lncRNAs termed *PAPAS* (promoter and pre-rRNA antisense) is derived from rDNA gene transcription in the antisense orientation [57]. During quiescence, *PAPAS* is upregulated to guide the H4K20 methyltrasnferase Suv4-20h2 to nucleolar chromatin, leading to increased trimethylation of H4K20 and chromatin compaction at rDNA loci [58].

***PTENpg1-AS***. The lncRNA *PTENpg1-AS*, which represses expression of the tumor-suppressor gene phosphatase and tensin homolog (PTEN), exists in two isoforms, α and β. *PTENpg1-ASα* represses PTEN expression by lowering its transcription via its interaction with DNMT3a and enhancer of Zeste on the *PTEN* promoter, while *PTENpg1-ASβ* regulates *PTEN* mRNA stability [59]. Given the role of PTEN in aging, senescence, and age-related diseases such as cancer, we anticipate a direct function for *PTENpg1-AS* in these processes [60-64].

***TARID.*** The lncRNA *TARID* (TCF21 antisense RNA inducing demethylation) has the ability to trigger expression of the tumor suppressor protein TCF21 (transcritpion factor 21) by inducing promoter demethylation. During this process, *TARID* binds to GADD45A (Growth arrest and DNA-damage inducible alpha), a regulator of DNA demethylation, and recruits it to the *TCF21* promoter. Interestingly, *TARID* is expressed in the antisense orientation to the *TCF21* gene [65].

#### Histone modifications

The most common epigenetic alterations during aging and senescence include enhanced histone H4K16 acetylation, H4K20 trimethylation and H3K4 trimethylation, and decreased histone H3K9 methylation and H3K27 trimethylation [66, 67, 68, 69]. Importantly, disruption of histone modification influences longevity in nematodes, flies, and worms [70-72]. In this section, we examine lncRNAs implicated in histone modifications, particularly methylation, that play a role in processes such as cell cycle progression, inflammation, and senescence.

***Kcnq1ot1***. The levels of the potassium channel protein KvLQT1 (encoded by the gene *KCNQ1*) decline with aging and age-related cardiovascular disease [73, 74]. The 91-kb long lncRNA *Kcnq1ot1* influences histone modifications by recruiting the histone methyl-transferases G9a and polycomb repressor complex (PRC2) to increase H3K9 and H3K27 trimethylation and repress transcription of the *Kcnq1* locus [75].

***NeST***. The lncRNA *NeST* (nettoie Salmonella pas Theiler's) binds to the H3K4 methyltransferase component WDR5 (WD repeat domain 5) and recruits it to the IFNγ locus. Given that lncRNA *NeST* is involved in inflammation during microbial infection [76] and that IFNγ methylation increases with advancing age, *NeST* could contribute to the inflammatory response and infection in elderly.

***ANRIL.*** The lncRNA *ANRIL* is transcribed from the same locus as *INK4b/ARF/INK4a* but in the opposite direction. *ANRIL* is involved in cell cycle regulation at least in part by recruiting CBX7 (Chromobox 7), a protein component of the of polycomb repressor complex 1 (PRC1), to the locus, increasing H3K27 methylation and thereby repressing *INK4a* transcription. Interestingly, CBX7 variants with point mutations that disrupt binding to RNA or methylated H3K27 repress genes in the *INK4a* locus, impairing cellular senescence [77].

***ANRASSF1***. The promoter of gene *RASSF1A*, which encodes the Ras association domain-containing protein 1, was found to be increasingly hypermethylated with advancing age [78]. The lncRNA *ANRASSF1* forms an RNA/DNA hybrid at the *RASSF1A* transcriptional start site and recruits the chromatin-modifying PRC2 complex to the *RASSF1A* promoter. The PCR2 complex then selectively modifies histone H3K27 methylation, reducing *RASSF1A* transcription [79].

***PINT.*** The p53-induced lncRNA *PINT* also interacts with PRC2 to regulate the expression of proteins in the TGF-β, MAPK and p53 pathways, which are associated with senescence, aging, and age-related diseases [2, 80-82].

#### Heterochromatin formation

Global disruption of heterochromatin is a hallmark of senescence and aging. In senescent cells, dramatic remodeling of the chromatin is associated with the formation of *s*enescence-*a*ssociated *h*eterochromatin *f*oci (SAHF) [83, 84]. The loss of heterochromatin with aging is believed to underlie various cellular processes associated with aging [85, 86]. Heterochromatin formation was found to promote longevity in *Drosophila* and the function of heterochromatin binding protein 1 (HP1a) correlates with life span in flies [87]. In addition, chromosomal stability is directly related to hetero-chromatin formation and maintenance. Epigenetic features including H3K9 trimethylation, H4K20 trimethylation, and HP1a binding are observed at constitutive heterochromatin in chromosomal DNA repeat regions such as subtelomeric and pericentromeric regions [88].

***TERRA***. Telomeric heterochromatin is modulated by *TERRA* through binding to several telomeric proteins: telomere repeat factors (TRF1, TRF2), origin recognition complex (ORC), HP1, and H3K9me3. Through these interactions *TERRA* helps to maintain telomeric structure and heterochromatin [89].

***BORDERLINE.*** This lncRNA was found to prevent spreading of the HP1 protein SWI6 and H3K9 methylation beyond the pericentromeric region in yeast. Interestingly, *BORDERLINE* is processed into short RNAs (*brdrRNAs*) by Dicer, a type-III RNase implicated in aging and senescence [90-92]. Accordingly, Dicer might modulate *BORDERLINE* levels, function, and processing to *brdrRNAs* in age-relevant processes.

### lncRNAs and proteostasis

Proteostasis (protein homeostasis) encompasses several biological processes that govern protein biogenesis, folding, trafficking, activity, interactions, degradation, and elimination. Disruption of proteostasis can lead to age-related diseases such as Alzheimer's, Parkinson's, and Huntington's diseases [93, 94]. Major proteolytic systems, including autophagy and the ubiquitin-proteasome pathway, decline with age, indicating that proteostasis is a common feature of aging [1]. In this section, we review the lncRNAs associated with proteostasis, including autophagy, and protein synthesis, trafficking, assembly, and degradation.

#### LncRNAs in autophagy

Changes in autophagy are another hallmark of aging [1]. Disruption of autophagy induces changes in mammalian tissue that resemble those associated with aging. Inhibition of autophagy also compromises the aging-inhibitory effects of interventions such as caloric restriction and sirtuin 1 activation [95]. Although their interdependence is not clearly understood, autophagy and senescence are integral processes of the cellular response to stress agents [96].

***HULC*, *MEG3,*** and ***7SL***. These lncRNAs modulate autophagy in different ways. Overexpression of *HULC* in SGC7901 human gastric cancer cells promoted cell proliferation and invasion, and inhibited apoptosis by inducing autophagy [97]. In contrast, the levels of *MEG3* and *7SL* correlated inversely with the level of the autophagy marker LC3-II *in vivo* [14, 98, 99]. The underlying mechanisms through which these lncRNAs influence autophagy are not well understood, but they may involve p53, since *7SL* can regulate *p53* mRNA translation and *MEG3* can regulate p53 directly via RNA-protein association or indirectly by lowering MDM2 expression levels [100, 101]. In sum, the aging- and senescence-associated process of autophagy is robustly affected by lncRNAs.

#### LncRNAs in protein trafficking

Some lncRNAs influence homeostasis by modulating the traffic of proteins and hence the abundance of a protein in a particular subcellular compartment. By recruiting transcription factors to the nucleus and specifically to certain DNA regions, lncRNAs can modulate transcription. Similarly, by recruiting certain RNA-binding proteins into ribonucleoprotein complexes, lncRNAs can elicit other gene regulatory functions. Although the complete spectrum of mechanisms through which lncRNAs affect protein trafficking and their impact on aging and senescence are largely unknown, a few examples are beginning to emerge.

***GAS5.*** This lncRNA induces growth arrest by acting as a decoy for the transcription factor glucocorticoid receptor (GR) and thus inhibits GR-mediated gene expression [102]. Specifically, *GAS5* binds GR in the cytoplasm and prevents its mobilization to the nucleus. Interestingly, the level of GR protein decreased in the nucleus but not the cytosol of aged Long-Evans rats with cognitive impairment [103], although it is not yet known if *GAS5* is directly involved in the aberrant GR distribution in this animal model of aging.

***PANDA***. The lncRNA *PANDA* is induced by p53 upon DNA damage. It binds the transcription factor NF-YA and interferes with its transcriptional activity, lowering the expression of apoptotic genes. Interestingly, NF-YA interaction with p53 impacts upon cell cycle regulation and senescence [104, 105]. These findings indicate that *PANDA* could be involved in DNA damage-induced senescence through NF-YA and p53.

***Gadd7***. In Chinese hamster ovary (CHO) cells, *Gadd7* was induced by DNA damage and oxidative stress and controlled the G1/S checkpoint and cell growth [106]. *Gadd7* associated directly with TDP-43 and interfered with the binding of TAR DNA-binding protein (TDP-43) to *Cdk6* mRNA, leading to *Cdk6* mRNA destabilization, abnormal cell cycle progression, and possibly senescence [107, 108]. It is not known if *Gadd7* interferes with other TDP-43 targets or if it influences other RNA-binding proteins involved in senescence or aging.

***7SL***. This highly abundant lncRNA interacts with the *TP53* mRNA and suppresses p53 translation. The RNA-binding protein HuR can displace *7SL* and enhances p53 translation. According to this competitive interaction, silencing *7SL* increased HuR binding to *TP53* mRNA and promoted p53 translation, in turn enhancing cell cycle arrest and senescence [14].

#### lncRNAs associated with protein synthesis and degradation

Protein synthesis and degradation are two critical processes that determine the steady-state abundance of proteins in cells. Protein synthesis is controlled by the rates of mRNA translation, while the ubiquitin proteasome pathway is the major driver for protein degradation. Some lncRNAs modulate protein levels indirectly by influencing the available pool of microRNAs and thereby affecting mRNA turnover and translation; for instance, lncRNAs such as *linc-MD1* and *lincRNA-RoR* (below) can act as decoys for miRNAs that usually suppress the translation or stability of other mRNAs [17, 109]. However, some lncRNAs interact directly with mRNAs to enhance or suppress their translation or with proteins to modulate their half-lives:

***AS Uchl1***. *AS Uchl1* enhances translation of UCHL1 (ubiquitin carboxy-terminal hydrolase L1) through an embedded SINE (short interspersed nuclear element) B2 repeat present in *AS Uchl1* [110]. UCHL1 is involved in brain development and age-related neurodegenerative pathologies such as Parkinson's disease [111]. Overexpression of UCHL1 was found to induce senescence, likely due to increased production of p14ARF, p53, p27KIP1 and decreased MDM2 levels [112]. Accordingly, *AS Uchl1* lncRNA may influence both senescence and neurodegeneration.

***LncRNA-p21***. Unlike *AS Uchl1*, the human *lncRNA-p21* was found to interact with target *CTNNB* and *JUNB* mRNAs (encoding β-catenin and JunB, respectively) and suppressed their translation by recruiting translation repressors RCK and FMRP [13]. The exact role of *lncRNA-p21* senescence and aging remains to be investigated, although β-catenin and JunB are known to influence cell proliferation and carcinogenesis [113-116].

***HOTAIR***. The levels of *HOTAIR*, a lncRNA upregulated in senescence, are reduced by the senescence-repressor HuR, an RNA-binding protein, via degradation of *HOTAIR* in a microRNA-dependent manner. *HOTAIR* was shown to serve as a scaffold to promote the ubiquitination and subsequent degradation of Ataxin-1 and Snurportin-1 [7]. These findings suggested that senescence-associated lncRNAs can function as platforms to facilitate protein ubiquitination and degradation to elicit cellular senescence.

#### LncRNAs and complex assembly

The assembly of protein complexes is widely required for the modulation of gene expression patterns. Several lncRNAs function in complexes with proteins, RNA, and DNA, as explained above. For instance, the lncRNA *TERC* is essential for the telomere complex formation that maintains telomere length [21, 117, 118], *H19* interacts with MBD1 to form a ribonucleoprotein complex that recruits histone lysine methyltransferases to suppress gene expression [36], and *ecCEBP* forms a complex with DNMT1 to regulate DNA methylation and silencing of the *CEBP* gene [48]. The lncRNA *pRNA* interacts with DNA at a site which is specifically recognized by the DNMT3b to regulate rRNA transcription [53], *PTENpg1-AS* lncRNA interacts with DNMT3a forming a ribonucleoprotein complex to regulate PTEN transcription [59], and lncRNAs *Kcnq1ot1*, *ANRIL*, *ANRASSF1*, and *PINT* form complexes with histones or histone modifiers to regulate gene expression as explained above. Other examples include lncRNA-protein complexes *NcRNA_CCND1_*-TLS, which are recruited to the promoter to suppress transcription of the *CCND1* gene following DNA damage [119], and *THRIL*-hnRNPL, which is recruited to the tumor necrosis factor (TNF)α promoter site to regulate TNFα expression [120]. These complexes broadly regulate gene expression programs relevant to the control of cell cycle progression and inflammation, two processes central to senescence and aging.

### LncRNAs and Stem cells

Stem cells are progenitor cells for the development of specific cell types in different tissues. The ability of stem cells to regenerate tissues (also known as ‘pluripotency’) declines with age. Like adult cells, stem cells are also exposed to stimuli that promote aging or senescence such as DNA damage and cell cycle inhibition. Disruption of stem cell gene expression programs is deleterious for various tissues and promotes age-related physiologic declines like immunosenescence (diminished production of adaptive immune cells) and reduction in muscle mass. They also underlie many pathologies, including cancer [1]. Pluripotency in human and mouse embryonic stem cells (ESCs) is tightly regulated and involves transcription factors (TFs) such as Oct4, Sox2 and Nanog along with several other coregulators [121]. These TFs transcribe genes that encode proteins involved in the maintenance of pluripotency and suppress the transcription of genes that encode proteins necessary for developing specific cell types [122]. Examples of lncRNAs that affect stem cell homeostasis are emerging, as discussed below.

#### Cross-regulation between lncRNAs and stem cell transcription factors

LncRNAs can regulate certain stem-cell transcription factors, which reciprocally can regulate the expression of lncRNAs. The abundance of some conserved lncRNAs is transcriptionally regulated by Oct4 and Nanog in mouse ESCs: *AK028326* is activated by Oct4, while *AK141205* is repressed by Nanog. Interestingly, knockdown or overexpression of these lncRNAs altered *Oct4* and *Nanog* mRNA levels and subsequently cellular lineage-specific gene expression and pluripotency, suggesting a feedback regulatory loop [123]. In mouse ESCs, several lncRNAs involved in stem cell gene regulation were identified that bound chromatin-regulatory proteins and coordinated gene expression programs [124].

***ES1, ES2, ES3***. The levels of the transcription factor REST are elevated in ESCs and decline as ESCs convert to neuronal SCs [125]. REST is involved in neurodegeneration, neurogenesis, and neuronal pathologies like ischemia, epilepsy, and Huntington's disease [126, 127]. REST has been suggested to regulate the expression of lncRNAs which could be involved in neurodegeneration and cancer [128]. A more detailed study of human lncRNAs that promote pluripotency and neuronal differentiation indicated that lncRNAs *ES1*, *ES2*, and *ES3* are exclusively expressed in human ESC or iPSC and also regulated by *Nanog* and/or *Oct4* [129]. Interestingly, depletion of these lncRNAs was proposed to inhibit neurogenesis through binding to the epigenetic regulators REST and SUZ12 [129].

***linc-RoR***. Recently, another report indicated that the expression of 28 lncRNAs was higher in iPSCs than in ESCs, suggesting that they might be required for the development of iPSCs. One of these lncRNAs, *linc-RoR*, was found to regulate the reprograming of hESCs. Silencing of *linc-RoR* inhibited the formation of iPSC colonies while its overexpression favored reprograming, prompting the hypothesis that *linc-RoR* might act as an endogenous miRNA sponge to regulate Oct4, Nanog, and Sox2 in hESCs [130]. As *linc-RoR* levels decrease rapidly under differentiation conditions, it was proposed to be a self-renewal and pluripotency marker for hESCs [109, 131]. *Linc-RoR was also found to lower the levels of the tumor suppressor and cell cycle regulator p53 under DNA damage conditions; similar to UCA1 (below), linc-RoR interacts with p-hnRNP I, which is required for p53 mRNA translation* [132]*. These findings indicate that linc-RoR is a potential ste*m cell regulator and modulator of cell growth and survival. The influence of *linc-RoR* on p53 expression connects *linc-RoR* to the control of cell cycle progression and cellular senescence.

#### Histone methylation regulates stem cell lncRNAs

Profiling of mouse ES cells differentiated into embryoid bodies identified 174 differentially expressed lncRNAs, many of which were associated with developmental genes (e.g., *Dlx1*, *Dlx4*, *Gata6*, *Ecsit*), including the pluripotency-associated lncRNAs *Evx1as* and *Hoxb5/6as*. They are derived from homeotic loci and associated with trimethylated H3K4 histones and the histone methyltransferase MLL1 [133]. Further studies suggested that lncRNA transcription in mouse ESCs is regulated by promoter methylation at CpG similar to protein-coding genes. For example, loss of the repressive H3K27me3 upon differentiation transcriptionally activated many lncRNAs, suggesting that ESC-specific lncRNAs are regulated via mechanisms similar to those that control protein-coding gene expression to influence stem cell differentiation and maintain pluripotency [134].

### Cell cycle-associated lncRNAs

Environmental stresses, telomere dysfunction, oxidative stress, and DNA damage negatively influence normal cell cycle progression causing cells to withdraw from cell cycle, halt replication and enter senescence. Thus, as tissues and organs age, terminally arrested senescent cells accumulate [135-137]. Accordingly, DNA damage, a suggested primary cause of cellular senescence and organismal aging, increases with advancing age and elevates cell cycle inhibitors (e.g., p53 and p21) that promote senescence [138]. Recent insight into lncRNAs involved in cell cycle regulation and senescence were mentioned earlier in this review. Here, we discuss cell cycle-related lncRNAs that may influence senescence and organismal aging.

***MALAT1.*** Several lines of evidence suggest that *MALAT1* represses senescence. For example, depletion of *MALAT1* in CaSki human cervical cancer cells induced G1 arrest and reduced cell growth, cell proliferation, and tumor size [139], and depletion of *MALAT1* in the breast cancer cell line MB231 decreased cell proliferation and triggered G1 arrest [140]. Similarly, silencing *MALAT1* in proliferating WI-38 cells enhanced senescence and induced G1/S arrest, two sets of effects that were linked to p53 actions [34, 141]. Together, these studies indicate that *MALAT1* might be essential for cell proliferation. Unexpectedly, however *MALAT1*-knockout mice lacked an obvious phenotype or histological abnormalities, indicating that *MALAT1* is not essential for development [142], although its impact may be revealed under specific pathological or environmental conditions.

***ANRIL***. As mentioned above, the lncRNA *ANRIL* is expressed from the p15/*CDKN2B*/*CDKN2A*/*ARF* gene cluster [143]. Downregulation of *ANRIL* significantly enhanced the levels of the cdk inhibitor (cdki) p15 and moderately increased the levels of cdki p16. Genome-wide analysis indicated that *ANRIL* levels decline in senescent WI-38 cells, and its silencing increased the abundance of cell cycle inhibitors and induced senescence [34, 144]. While these findings indicate that *ANRIL* is required for cell cycle progression and suppression of senescence, further studies are warranted to understand the roles of *ANRIL* more broadly in aging.

***NcRNA_CCND1_***. The CCND1-associated lncRNA *NcRNA_CCND1_* modulates the expression of the cell cycle regulator cyclin D1 (CCND1), which is required for the activity of cdks, particularly Cdk2 and cdk4, to progress through the G1/S phase transition [145]. Upon exposure to DNA-damaging agents, the *NcRNA_CCND1_* binds the RNA-binding protein TLS to form a ribonucleoprotein complex that is recruited to the *CCND1* promoter and inhibits transcription [119]. Since CCND1 is a major regulator of the cell cycle progression, *NcRNA_CCND1_* will likely be implicated in aging-relevant processes such as cancer.

***SRA.*** *SRA* is a co-activator of steroid receptors and other transcription factors such as the myogenic regulatory transcription factor MyoD and the major regulator of adipogenesis PPARγ [146, 147]. Overexpression of *SRA* promotes the differentiation of mesenchymal precursor cells into adipocytes; conversely, *SRA* knockdown inhibits preadipocyte differentiation. *SRA* decreases the expression of cdkis p21 and p27 and it increases phosphorylation of Cdk1 [146, 147]. Since *SRA* is expressed in a wide range of tissues [148], similar regulatory mechanisms may occur in a variety of tissues that impact upon cell cycle progression, senescence, and adipogenesis.

***HEIH.*** The lncRNA *HEIH* is highly expressed in human Hepatitis B virus-related hepatocellular carcinoma (HCC). It suppresses the expression of cdkis p16, p21, p27, and p57 and hence facilitates tumor cell growth [149]. Future studies are warranted to study the role of *HEIH* lncRNA in aging.

***HULC.*** The lncRNA *HULC* is highly expressed in hepatocytes, HCC, and hepatic colorectal carcinomas [150, 151]. Hepatitis B virus X protein induces CREB-mediated *HULC* expression, which subsequently promotes hepatoma cell proliferation by lowering the expression of the cdki p18 [152, 153]. Thus, *HULC* may play a role in cell cycle progression and cellular senescence.

***Gadd7***. As mentioned above, *Gadd7* was upregulated by DNA damage [106]. *Gadd7* decreases cell growth by binding TDP-43 and preventing its interaction with *Cdk6* mRNA, leading to *Cdk6* mRNA decay [107]. As CDK6 affects cell cycle and senescence [108, 154], it is expected to influence mammalian aging.

***UCA1.*** *UCA1* is highly expressed in bladder transitional cell carcinoma [155]. Recently, *UCA1* was found to promote breast cancer cell growth by lowering the expression of the tumor suppressor and cdki p27. Silencing *UCA1* enhanced hnRNP I abundance in the cytoplasm, facilitating *p27* mRNA translation and triggering G1 cell cycle arrest [156]. These findings suggest that *UCA1* can affect cellular senescence and carcinogenesis by modulating p27 levels [157].

***eRNAs***. Enhancer ncRNAs (*eRNAs*) are short (50-1500 bp) RNAs transcribed from certain enhancer regions to influence transcription at distant sites (up to 1 Mbp away) [158-160]. Binding of p53 to enhancer regions (p53BERs) produced p53-dependent *eRNAs* that modulated p53 transcriptional activity and induced p53-dependent cell cycle arrest [161], linking eRNAs influence to senescence, aging, and carcinogenesis.

***H19.*** As mentioned above, *H19* is an epigenetic regulatory RNA that affects cell growth and proliferation. In breast cancer cells, *H19* knockdown decreased cell growth and formation of colonies in culture [162]. Hypoxia elevated *H19* abundance in HHC cells and *H19* knockdown significantly reduced tumor growth after recovery following hypoxia. Additionally, *H19* overexpression enhanced tumor growth in Hep3B cells [163]. These findings indicate that the high levels of *H19* in cancer cells led to enhanced growth and proliferation, as well as to the avoidance of cellular senescence. Indeed, H19 downregulation lowered the levels of p57, which participates in cell division, differentiation, cell survival, cell proliferation and tumorigenesis [39, 164-167].

***MEG3.*** The lncRNA *MEG3* is repressed in many human cancer cell lines due to gene deletion or to DNA hypermethylation. Forced expression of *MEG3* inhibited the growth of human cancer cell lines such as HeLa, MCF-7, and H4, suggesting that *MEG3* may act as a tumor suppressor [168], while downregulation of *MEG3* enhanced autophagy, increased cell proliferation and inhibited cell death [99]. A major mechanism through which *MEG3* regulates cell proliferation is by lowering the levels MDM2, a repressor of p53 [169]. *MEG3* expression also induces apoptosis and growth arrest in cervical cancer cells [170]. Global analysis on transcriptional networks and cellular senescence in human mammary fibroblasts suggested that the NR4A3 (nuclear receptor subfamily 4, group A, member3) is upregulated in senescent cells and affects cell growth via *MEG3* [171], further linking *MEG3* to cellular senescence and carcinogenesis.

***7SL***. Unlike *MEG3*, *7SL* lncRNA is highly expressed in cancer cells [14, 172]. *7SL* silencing was found to promote cell cycle arrest and senescence in HeLa cells. These effects were mediated, at least in part, through binding to and suppressing *TP53* mRNA translation by competing with the RNA-binding protein HuR, as explained above [14].

### LncRNAs and intercellular communications

Factors associated with aging in one tissue can be secreted, transported, and received by other tissues. These intercellular communication events involve the sharing of factors such as inflammatory cytokines and extracellular vesicles, which are associated with senescence, aging-associated declines, and diseases of advancing age [1, 173, 174].

#### Inflammation-associated lncRNAs

Senescent cells actively secrete pro-inflammatory cytokines, a phenomenon known as the senescence-associated secretory phenotype (SASP) [1, 175]. Given that senescent cells accumulate in older tissues, they are believed to be directly responsible for the low-grade pro-inflammatory state that characterizes aging. The emerging role of lncRNAs in inflammation is discussed here.

***17A***. The lncRNA *17A* is upregulated in cerebral tissues derived from Alzheimer disease patients as well as in response to inflammatory stimuli such as IL-1α. Interestingly, *17A* is encoded within the G-protein-coupled receptor 51 (*GPR51*) gene and *17A* overexpression enhanced secretion of Aβ and regulated GABA B alternative splicing and signaling [176]. In light of the fact that GPR function is associated with age-associated declines [177], *17A* provides an interesting link between GPRs and age-associated neurodegeneration.

***Lethe***. In mouse embryonic fibroblasts (MEFs), 54 lncRNAs were found to be regulated following treatment with TNFα, a pro-inflammatory cytokine associated with inflammation, aging, age-related diseases, and cellular senescence [178, 179]. Among these lncRNAs, *Lethe* was found to be particularly important for the pro-inflammatory state of aging tissues by providing an key negative feedback loop: binding of *Lethe* to the NF-κB subunit RelA inhibited DNA binding and reduced the production of inflammatory proteins [178].

***THRIL***. TNFα was also found to be induced by lncRNA *THRIL* (TNFα- and hnRNPL-related immunoregulatory lincRNA). This lncRNA interacts with hnRNP L, forming a ribonucleoprotein complex that binds the TNFα promoter and regulates its expression in THP1 macrophages [120]. Together, lncRNAs *lethe* and *THRIL* are involved in inflammation in a TNFα-dependent manner.

***Lnc-IL7R***. Recently, *Lnc-IL7R* was identified as a regulator of the lipopolysaccharide (LPS)-induced inflammatory response. Depletion of *Lnc-IL7R* reduces trimethylation of histone H3 at lysine 27 (H3K27me3) causing a reduction in the levels of inflammatory mediators including E-selectin, VCAM-1, IL-6 and IL-8 [180]. Given that pro-inflammatory cytokines are secreted by senescent cells and contribute to the aging phenotype [181, 182], *Lnc-IL7R* might be involved in sense-cence or aging by regulating these inflammatory factors.

**Other lncRNAs**. Toll-like receptors (TLRs) are vital in the response of macrophages to pathological stimuli. In normal aging, defects in TLR signaling enhanced inflammation [183]. Recently, inflammatory lncRNA signatures were characterized by RNA sequencing in healthy individuals treated with low-dose LPS, which activates TLR4 signaling. The study revealed tissue-specific regulation of distinct lncRNAs in the inflammatory physiology and pathology of the cardiovascular system [184]. However, the impact of these lncRNAs on age-related processes has not been investigated directly.

The TGF-β/Smad3 pathway is involved in inducing and resolving the inflammatory response [185]. High-throughput RNA analysis was used to identify Smad3-dependent lncRNAs related to renal inflammation. The analysis identified numerous lncRNAs altered in Smad3 knockout mice [186]. As this pathway is impaired with age and Smad3 regulates senescence phenotype [187, 188], this network of proteins and lncRNAs differentially expressed may play a role in aging-associated inflammation (‘inflammaging’).

#### LncRNAs and extracellular signaling through exosome

Like inflammatory cytokines, RNAs can serve as extracellular signaling molecules. RNAs can be transferred between cells through gap junctions or via extracellular vesicles such as exosomes and microvesicles [189]. Due to their presence in all body fluids, exosomes are believed to participate in many biological processes, including those that directly affect aging [173]. All RNAs, including mRNAs, dsRNAs, and miRNAs, can be transported among cells [190, 191]. The examples below highlight emerging functions of lncRNAs in intercellular communication in age-relevant processes, particularly inflammation and growth inhibition.

***TUC339***. Among the lncRNAs found in extracellular vesicles from hepatocellular carcinoma cells, the lncRNA *TUC339* modulates cell cycle progression, tumor growth, and adhesion [192, 193]. Since *TUC339* can be transported to distant tissues via extracellular vesicles, it can serve as a messenger to modulate the cell division cycle in distant tissues.

***Tie-1as***. Also involved in cell-cell communication, the lncRNA *tie-1as* interacts with and represses the mRNA encoding for tyrosine kinase-containing immunoglobulin and epidermal growth factor homology domain-1 (Tie-1), causing defective endothelial cell contact junctions [194]. Although endothelial function declines with age, the impact of *Tie-1as* on aging has not been examined directly.

***Linc-ROR***. This lncRNA is highly enriched in extracellular vesicles derived from hepatocellular carcinoma cells. TGFβ regulates the levels of *Linc-ROR* in the vesicles and incubation of vesicles with tumor cells reduces chemotherapy-induced cell death in recipient cells. Suppression of *Linc-ROR* leads to increased chemotherapy-induced apoptosis and cytotoxicity, suggesting that this lncRNA is a modulator of chemoresistence in hepatocellular carcinoma cells [195].

**Other lncRNAs**. Recently, several lncRNAs that are typically present in low abundance in cells were found to be selectively enriched in secreted exosomes, including *MALAT1, HOTAIR, LincRNA-p21, GAS5, TUG*1 and *CCND1-ncRNA*. Some of these lncRNAs are discussed above, as they influence cell cycle regulation, cellular senescence, and other processes. Treatment with the DNA damaging agent bleomycin elevated the concentration of *LincRNA-p21* and *ncRNA-CCND1* in exosomes [196]. Since damaged DNA accumulates with advancing age, the accumulation of these lncRNAs in exosomes may serve as a marker for aging in tissues.

### Concluding remarks and perspectives

Aging traits are governed through changes in subsets of expressed proteins. LncRNAs can modulate protein expression patterns by controlling gene transcription, mRNA stability, and protein abundance. Through this influence, lncRNAs modulate key molecular events underlying the aging process, including those discussed here – the control of telomere length, epigenetic gene expression, proteostasis, stem cell function, intercellular communication, cell proliferation and cellular senescence.

The examples discussed in this review underscore the growing recognition that lncRNAs critically affect both the physiologic decline that occurs with aging and the pathologies associated with advancing age. Accordingly, there is mounting interest in the diagnostic, prognostic, and therapeutic value of lncRNAs. The facile detection of lncRNAs in body fluids (e.g. exosomes) and the ease of design of molecules to increase or decrease lncRNA levels make them particularly attractive clinical targets.

However, the potential usefulness of lncRNAs in aging dysfunction and disease cannot be fully realized at present. First, we must have a more comprehensive understanding of age-associated lncRNAs, their spatiotemporal pattern of expression, the molecules with which they interact (proteins, DNA, and RNA), and the impact of altering their abundance upon cell function. Second, we need to develop suitable animal models in which we can study age-associated lncRNAs, assess their function in tissues, organs, and systems, and evaluate how they influence the aging process. By advancing in these areas of knowledge, we expect to gain a deeper molecular understanding of aging and develop more effective interventions to ameliorate the losses of advancing age.
